# Molecular Assemblies of Porphyrins and Macrocyclic Receptors: Recent Developments in Their Synthesis and Applications

**DOI:** 10.3390/molecules171011763

**Published:** 2012-10-09

**Authors:** Mickey Vinodh, Fatemeh H. Alipour, Abdirahman A. Mohamod, Talal F. Al-Azemi

**Affiliations:** Chemistry Department, Kuwait University, P.O. Box 5969, Safat 13060, Kuwait

**Keywords:** porphyrin, cyclodextrin, calixarene, resorcinarene, molecular recognition, host-guest systems

## Abstract

Metalloporphyrins which form the core of many bioenzymes and natural light harvesting or electron transport systems, exhibit a variety of selective functional properties depending on the state and surroundings with which they exist in biological systems. The specificity and ease with which they function in each of their bio-functions appear to be largely governed by the nature and disposition of the protein globule around the porphyrin reaction center. Synthetic porphyrin frameworks confined within or around a pre-organized molecular entity like the protein network in natural systems have attracted considerable attraction, especially in the field of biomimetic reactions. At the same time a large number of macrocyclic oligomers such as calixarenes, resorcinarenes, spherands, cyclodextrins and crown ethers have been investigated in detail as efficient molecular receptors. These molecular receptors are synthetic host molecules with enclosed interiors, which are designed three dimensionally to ensure strong and precise molecular encapsulation/recognition. Due to their complex structures, enclosed guest molecules reside in an environment isolated from the outside and as a consequence, physical properties and chemical reactions specific to that environment in these guest species can be identified. The facile incorporation of such molecular receptors into the highly photoactive and catalytically efficient porphyrin framework allows for convenient design of useful molecular systems with unique structural and functional properties. Such systems have provided over the years attractive model systems for the study of various biological and chemical processes, and the design of new materials and molecular devices. This review focuses on the recent developments in the synthesis of porphyrin assemblies associated with cyclodextrins, calixarenes and resorcinarenes and their potential applications in the fields of molecular encapsulation/recognition, and chemical catalysis.

## 1. Introduction

Nature shows a magnificent example of porphyrin usefulness, whereby this basic macrocyclic framework nature is able to perform the most essential life processes- activate and/or transport molecular oxygen in animals and convert sunlight in plant photosynthetic systems. The selectivity and specificity exhibited by pophyrins in each of their biological forms confirms it to be greatly influenced by the nature of the protein network around the porphyrin center [1,2,3]. Nowadays investigation of biomimetic reactions is very intense and a variety of synthetic porphyrins and their metalloderivatives have been demonstrated to be efficient model systems for many life processes and enzyme actions [4,5,6,7,8,9,10]. The special features of porphyrin include rigid, planar geometries, high stability at elevated temperatures or pH variations, easy oxidation and reduction, inherent symmetry, easily tunable electronic and redox properties and excellent photosensitization. The porphyrin macrocyle is a versatile platform for additional substitutions and construction of pre-organized functional materials based on macrocyclic porphyrin entities is a fascinating field of research [11,12,13,14,15,16,17,18]. Such intriguing architectures, beside their intrinsic intellectual stimuli, is of importance in many fields of chemistry and technology, such as material chemistry, catalysis, and light harvesting and sensor applications.

In this context, the new millennium has witnessed tremendous development in the generation of some novel porphyrin systems by incorporating into them some suitable foreign host material, called molecular receptors. Molecular receptors are macrocyclic, three-dimensional host molecules with guest inclusion capabilities to ensure strong and precise molecular recognition [19,20,21,22,23,24,25,26,27,28,29]. Molecular recognition using synthetic compounds is a process where some molecules (hosts) selectively bind other molecules or ions (guests) to produce a well structurally organized system through intermolecular forces and to mimic the mode of action of a natural enzyme by catalyzing specific reactions at ambient conditions. Most commonly the host is a concave organic molecule with an enclosed interior—the molecular cavity—large enough to selectively bind ionic or small molecular substrates (guests) by means of various intermolecular interactions. Host-guest chemistry is the simplest form of supramolecular chemistry. In complex formation and self-assembly, host-guest systems utilize noncovalent interactions, which include hydrogen bonds, hydrophobic/hydrophilic effects, aromatic π character and van der Waals interactions. Host-guest encapsulation can be achieved both in solution as well as in the solid state since dissolution of the host does not result in disappearance of the cavity. Due to their complex structures, enclosed guest molecules reside in a molecular cavity is at a different environment isolated from the outside. As a consequence, physical properties and chemical reactions specific to the environment can be identified. Such characteristics are similar to those of the protein tertiary structure of natural enzymes. Therefore, it is obvious that the molecular receptors could appropriately tune the reactivity of an attached porphyrin/metalloporphyrin just like all activities of natural porphyrins are controlled by the surrounding protein globule in biosystems.

Macrocyclic oligomers such as calixarenes, resorcinarenes, spherands, cyclodextrins and crown ethers are found to be attractive molecular host systems possessing excellent recognition ability. Construction of novel ensembles comprising porphyrins and such macrocyclic cavitants is one of the efficient strategies for enzyme-like molecular recognition. The combination of the unique catalytic and photosensitizing efficiency of metalloporphyrins with the receptor characteristics of these cavitants for selected species is basic foundation for such molecular recognition. Last two decades have witnessed highly active research work on this concept and achieved much progress in the synthesis and applications of porphyrin-cavitant systems.

In this review we attempt to cover the developments in the synthesis and application of porphyrin-attached molecular receptors which have appeared in the last 5 years. We are concentrated only on three major 3-dimensional molecular host materials—cyclodextrins, calixarenes and resorcinarenes. Different modes of incorporation of porphyrins into these cavitants are discussed, which include porphyrins covalently bonded to the cavitants, electrostatic self-assembly of charged porphyrins with cavitants and porphyrins encapsulated within the macrocyclic cavities.

## 2. Cyclodextrin-Porphyrin Assemblies

**Figure 1:**
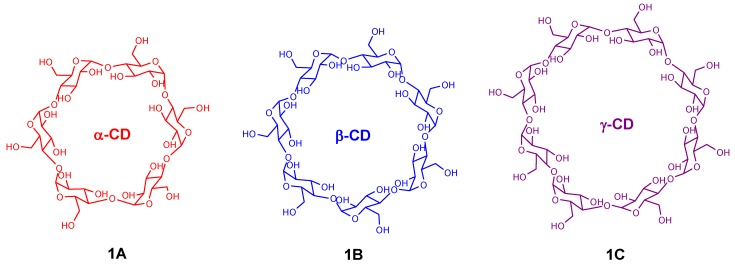
Structures of α-cyclodextrin (**1A**), β-cyclodextrin (**1B**) and γ-cyclodextrin (**1C**).

The cyclodextrins (CDs) are a family of cyclic oligosaccharides linked by α-1,4-glucopyranose units. The family is made up of three major and well-known CDs: α-, β-, and γ-CD, containing six (**1A**), seven (**1B**) and eight (**1C**) glucose subunits, respectively (Figure 1). Cyclodextrins feature a hydrophobic inner cavity and a hydrophilic outer surface with their shape resembling a truncated cone. CDs are extensively used not only as excellent receptors for molecular recognition but also as sophisticated building blocks for supramolecular architectures. The unique applications of cyclodextrins are mainly due to (i) their high water solubility; (ii) their ability to accommodate a wide variety of guest molecules and (iii) their well-defined molecular structure [21,22,23,24,25,30,31,32,33,34].

The binding interactions between porphyrins and cyclodextrins have provided novel molecular assemblies. Porphyrin-cyclodextrin interactions can be of different types: mutual bonding between them through ionic or covalent bonds or just simple encapsulation of the porphyrin into a CD cavity without any bonding interaction. After binding with CDs, some dynamic and static properties of porphyrin molecules, namely physical, photophysical, and photochemical properties, can change dramatically, and can cause changes in the equilibrium between monomer and assembled aggregates. All unique properties of CDs together with the catalytic and photosensitizing efficiency of porphyrin macrocycle have made the CD-porphyrin ensembles precious functional materials.

**Figure 2:**
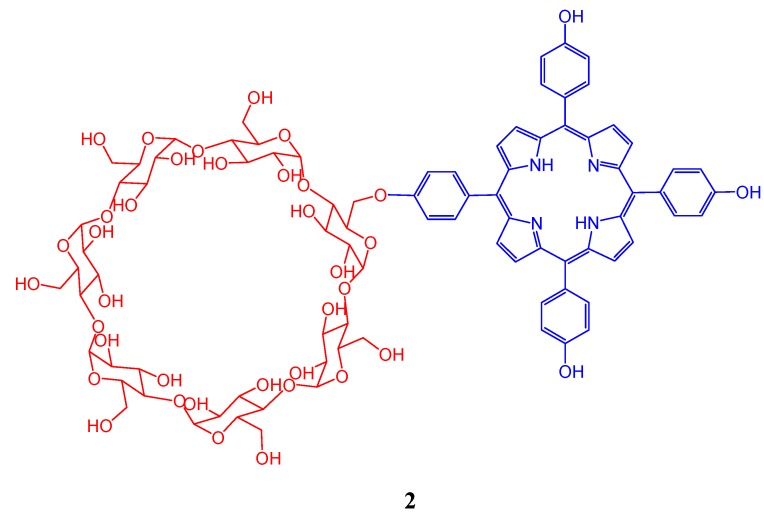
Schematic representation of 5[4-(6-*O*-β-cyclodextrin)-phenyl],10,15,20-tris(4-hydroxyphenyl)-porphyrin.

The covalent attachment between functionalized cyclodextrins with suitable porphyrin units are an attractive strategy to build up novel host-guest systems. A novel porphyrin-cyclodextrin conjugate, 5[4-(6-*O*-β-cyclodextrin)-phenyl],10,15,20-tris(4-hydroxyphenyl)-porphyrin (CD-THPP) (Figure 2), has been synthesized by Puglisi *et al*. in good yield starting from 6-*O*-tosyl-β-cyclodextrin using 5,10,15,20-tetra(4-hydroxyphenyl)porphyrin (*p*-THPP) as a nucleophile [35]. In neat methanol, CD-THPP is found to be present in monomeric form and exhibits spectroscopic features virtually identical to that of *p*-THPP. In water-methanol solvent mixture (9:1; v/v), CD-THPP shows a tendency to self-arrange as a dimer, whose formation is strongly encouraged by stacking interactions between the aromatic rings and intermolecular hydrophobic porphyrin-cyclodextrin interactions. This supramolecular species exhibited a strong exciton coupling and, contrary to the parent *p*-THPP, showed a good response to light excitation, as confirmed by the considerable fluorescence emission and triplet-triplet transient absorption. The authors also reported that incorporation of suitable guests into the CD cavity triggers a new rearrangement of the dimer with a higher degree of overlap between the porphyrin chromophores, facilitating the occurrence of intramolecular photoinduced electron transfer between the guest and the porphyrin unit.

**Figure 3:**
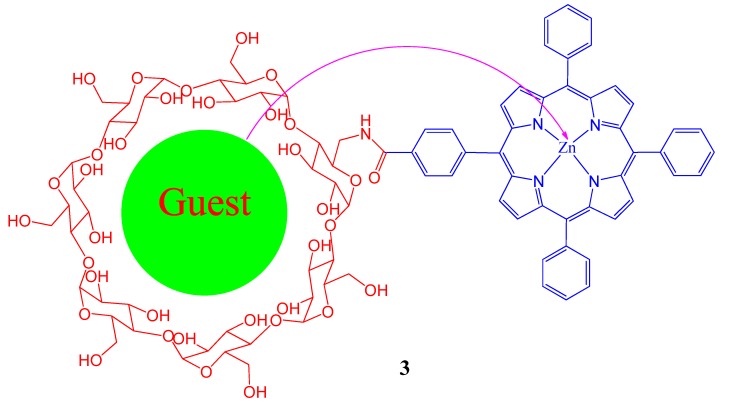
Energy transfer from the guest species to metalloporphyrin of the cyclodextrin-porphyrin conjugate.

It is reported that covalent attachment of a porphyrin head group onto a guanine-rich oligonucleotide strand can enhance the self-assembly of DNA quadruplexes through porphyrin-based π-π interactions [36]. Modulation of these allosteric interactions is found to be possible by addition of a porphyrin-cyclodextrin complexing derivative which allows a high degree of control over the formation and disassembly of these guanine quadruplexes. A composite of Zn tetraphenylporphyrin linked with β-cyclodextrin (Figure 3), was prepared as a convenient scaffold for a self-assembled energy-transfer complex and the energy-transfer properties of this host-complex were studied for several organic guests by fluorescence and fluorescence-excitation spectroscopies [37].

**Figure 4:**
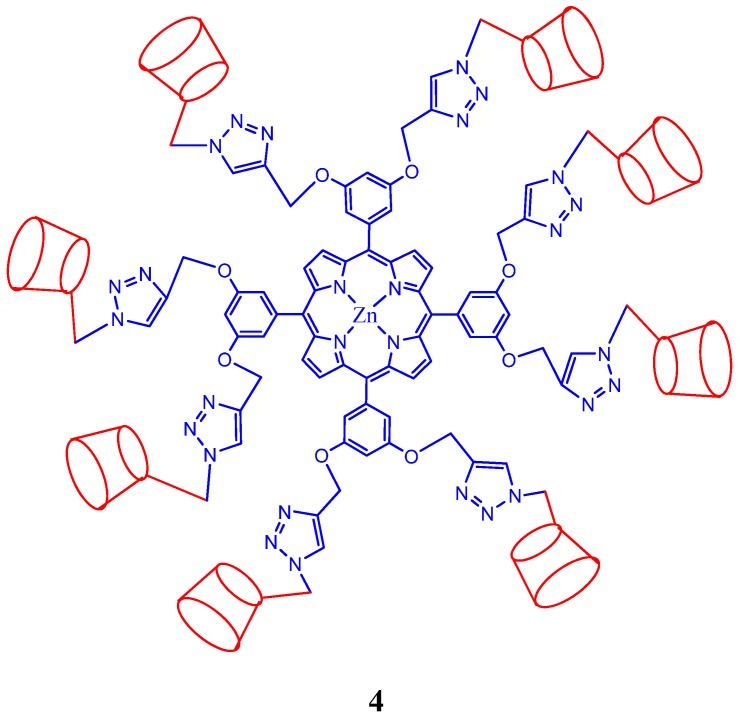
Schematic representation of cyclodextrin-Zinc porphyrin system.

A novel water soluble nano-rod, synthesized by self-assembly of pristine C_60_ and a double-sided porphyrin projecting four β-cyclodextrins from each face, as shown in Figure 4, has been reported by Fathalla *et al*. [38].

**Figure 5:**
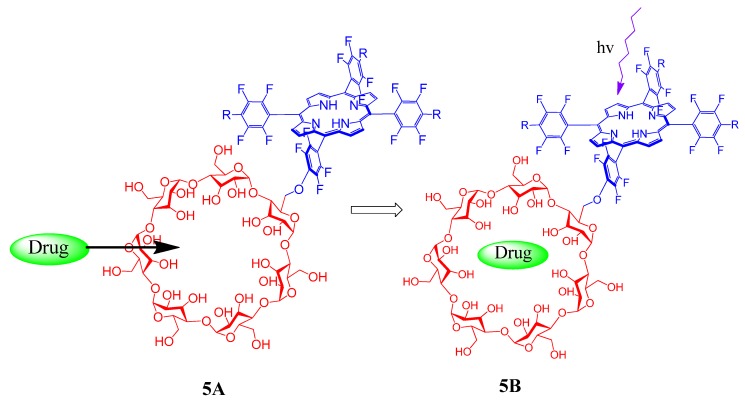
Schematic representation of combined chemotherapy and photodynamic therapy achieved by a drug encapsulated cyclodextrin-porphyrin conjugate (**5B**).

The most fascinating area of cyclodextrin-porphyrin system is in the field of drug delivery and related biomedical applications. Many porphyrin-cyclodextrin conjugates were prepared and tested for selective and effective multifunctional drug delivery and therapy. A porphyrin-CD system **5A** (Figure 5) in which efficient binding of the selected drug to the cyclodextrin cavity and photosensitizing properties of the porphyrin moiety causes photodamage to the cancer tissues is described by Kralova *et al*. [39]. This combined effect of chemotherapy and photodynamic therapy (compound **5B**) has been demonstrated by various *in vitro* and *in vivo* studies.

**Figure 6:**
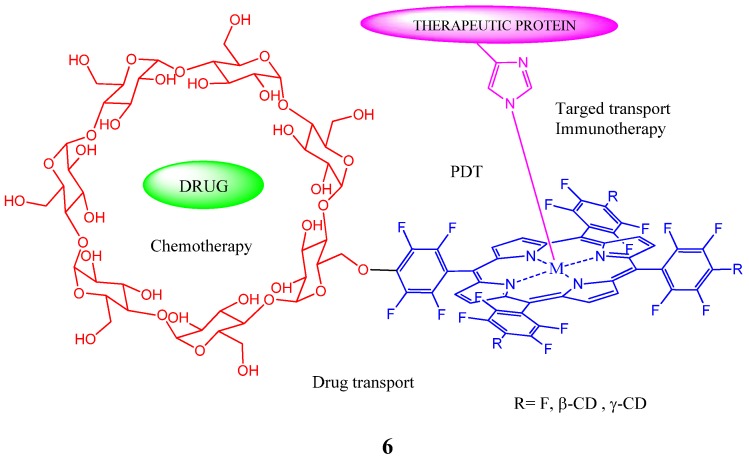
Therapeutic protein coordinated drug delivery system (**6**).

The coordination attachment of therapeutic proteins to a drug delivery system consists of cyclodextrin conjugated metalloporphyrin **6** (Figure 6), and its application in combined therapy is demonstrated by Kejik *et al*. [40].

Proof of concept is described by the synthesizing a drug delivery system for cytostatics, which is based on a combination of Zn-porphyrin-cyclodextrin conjugates and their supramolecular coordination complexes with various therapeutic proteins. This system allows combined photodynamic therapy, cell targeted chemotherapy and immunotherapy. Compared to the efficiency of building blocks used for the construction of the system, this coordination assembly exhibited therapeutic superiority, when tested in a mouse model with human C32 carcinoma.

Supramolecular materials, made up of cyclodextrin-porphyrin combinations generated via the intermolecular non-covalent association of monomeric building blocks by the “bottom up” approach, have become highly active research area in the field of nanosynthesis, pharmaceutical and biomimetic applications. As discussed earlier, cyclodextrins, which are excellent receptors for molecular recognition are convenient building blocks to construct nanostructured supramolecular materials especially bioactive and environment sensitive ones. On the other hand, porphyrins particularly ionic ones are widely used in photodynamic therapy (PDT) and have very important applications as functional materials, in the oxygen transport and activation in biological processes. Therefore, the combination of CD and ionic porphyrin in a supramolecular network may bring a breakthrough in many fields of material science, biological/medicinal chemistry owing to several inherent advantages. For example, the strong association of the CD cavity with anionic porphyrin in water can not only prevent the unfavorable porphyrin-porphyrin aggregation, but also enable the high stability of CD/porphyrin system in the biological environments such as body fluid. The presence of cyclodextrin also protects the porphyrin component in its transportation to the appropriate cellular site without undesirable damage.

**Figure 7:**
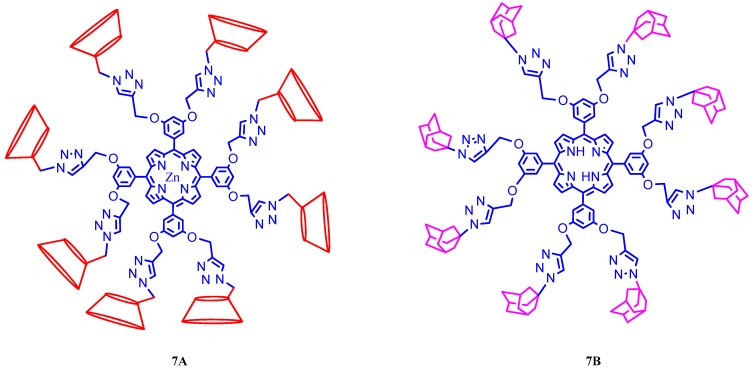
Porphyrin-cyclodextrin conjugate (**7A**) and porphyrin-adamantane conjugate (**7B)** employed to generate porphyrin nanowares.

Supramolecular 1:1 inclusion complexes of 5-(*p*-hydroxyphenyl)-10,15,20-tris-(4-chlorophenyl)-porphyrin (*p*-HTClPP) with four different types of cyclodextrins, namely β-cyclodextrin (β-CD), heptakis(2,3,6-tri-*O*-methyl)-β-CD (TM-β-CD), carboxymethyl-β-cyclodextrin (CM-β-CD) and thio- butylether-β-CD (SBE-β-CD) were studied by absorption, fluorescence and ^1^H-NMR spectroscopy. The formation of inclusion complexes was confirmed by observing the changes of spectroscopy properties [41]. Compared with parent native β-CD, the inclusion abilities of modified β-CDs with *p*-HTClPP are found to be stronger. The hydrophobic effect plays an important role in the inclusion procedure. Interesting variations in fluorescence intensity of the *p*-HTClPP is also observed in these inclusion complexes. A complex of tetrakis(4-hydroxylphenyl)porphyrin (THPP) and cyclodextrin-modified multi-walled carbon nanotubes (MWNTs-CD) was prepared by Zhang and coworkers [42]. The electron distribution of THPP is found to not be influenced by MWNTs-CD as evidenced by the UV-Vis spectra. On the other hand, the fluorescence of THPP is quenched, suggesting that there exists energy transfer between THPP and MWNTs-CD. The electrochemical behavior of porphyrin complex is similar to that of the inclusion complex of amino-modified cyclodextrin and THPP, but different from that of the MWNTs-CD. These phenomena indicate that the cyclodextrin moieties play an important role in the interaction between THPP and MWNTs-CD. It is also reported that self-assembly of porphyrin-CD conjugates **7A** (Figure 7) and porphyrin-adamantane conjugates **7B** in water yielded well-defined stable porphyrin nanowires wherein the individual monomers do not aggregate via π-π interactions [43].

Interaction of *meso*-tetrakis(2-thienyl)porphyrin (H2TTP) and its Cu-derivative (Cu-TTP) with α-cyclodextrin (α-CD), β-CD, γ-CD, heptakis(2,3,6-tri-*O*-methyl)-β-CD (TM-β-CD) was studied in phosphate buffer [44]. The H2TTP and Cu-TTP can form 1:2 inclusion complexes with TM-β-CD and 1:1 inclusion complexes with the other three cyclodextrins. Also the inclusion ability of α-CD with H2TTP and Cu-TTP is the strongest among the three native CDs. The inclusion ability of TM-β-CD with H2TTP and Cu-TTP is stronger, compared to the native β-CD, which indicates that the capacity matching plays a crucial role in the inclusion procedure except for the hydrophobic effect.

A porphyrin ([([5,15-bis(4-(3-(1*H*-pyrrol-1-yl)propoxy)phenyl)-10,20-diphenylporphinato]zinc(II)) film on an indium-tin oxide (ITO) electrode was prepared in which the self-aggregation of the porphyrin film was suppressed by encapsulation of the porphyrin unit in the cavity of cyclodextrin [45]. The photocurrent density and the quantum yield in this porphyrin-cyclodextrin system are found to be improved and it was demonstrated that this high quantum yield, perhaps 25 times larger, arises from the isolation of the porphyrin unit by cyclodextrin through host-guest interactions.

The interaction of 5-pyridyl-10,15,20-tris-(*p*-chlorophenyl)porphyrin (PyTPP) with β-CD and heptakis(2,3,6-tri-*O*-methyl)-β-CD (TM-β-CD) were studied by Guo *et al*. [46]. Compared to β-CD, the inclusion ability of TM-β-CD with PyTPP is stronger. PyTPP also prefers to form the 1:1 inclusion complex with TM-β-CD, but hardly forms inclusion complex with β-CD. The interaction of PyTPP with DNA was further also carried out in the presence and absence of TM-β-CD. In the presence of TM-β-CD a significant decrease of the binding constant and binding numbers were observed and the interaction of porphyrin-bound DNA has been inhibited, which was due to the fact that PyTPP enter into the cavity of TM-β-CD and influence binding affinity of PyTPP to DNA. The aggregation behavior of the zinc porphyrin-β-CD conjugate in a water/ethanol binary mixed solution was investigated [47]. The spectroscopic data and the atomic force microscopy (AFM) image strongly suggest that a part of a Zn porphyrin is included in the β-CD nanocavity of another ZnP-β-CD conjugate at certain concentrations and hence leading to the formation of Zn porphyrin J-aggregates.

A novel multiple supramolecular assembly fabricated through non-covalent interactions was reported recently, in which a folic acid-modified β-cyclodextrin acted as a target unit, an adamantanyl porphyrin acted as a linker unit, and graphene oxide acted as a carrier unit [48]. In this system, the graphene oxide unit could associate with the anticancer drug doxorubicin through π-π interactions, and the folic acid-modified β-cyclodextrin unit could recognize the folic acid receptors in cancer cells. Due to the cooperative contribution of these three units, the resulting supramolecular assembly, after association with doxorubicin, exhibited better drug activity and much lower toxicity than free doxorubicin *in vivo*.

**Figure 8:**
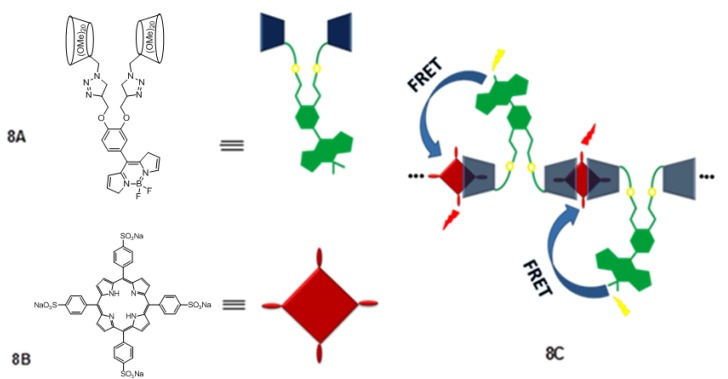
Representation of fluorescence resonance energy transfer (FRET) from modified cyclodextrin donor to porphyrin acceptor.

Many research groups are interested in the synthesis of inclusion complexes formed between anionic or cationic porphyrins with suitable cyclodextrins. 4,4-Difluoro-4-bora-3a,4a-diaza-s-indacene-bridged bis(permethyl-β-cyclodextrins) {3,4-[β-CD-triazole-CH_2_O]_2_C_6_H_3_}-BODIPY, (Figure 8, **8A**) was synthesized by Liu and coworkers through click chemistry, and it forms a well-defined linear assembly with 5,10,15,20-tetrakis(4-sulfonatophenyl)porphyrin (Figure 8, **8B**) via extremely strong host-guest interactions, (*i.e*. Figure 8, **8C**) [49]. This host-guest system exhibited highly efficient fluorescence resonance energy transfer (FRET) from BODIPY donor to porphyrin acceptor and is observed that the energy transfer quantum yield is as high as 94% by virtue of such a noncovalent path. BODIPY was employed as an energy donor because it has strong UV absorption that emits a relatively sharp fluorescence peak with high quantum yield, and is relatively insensitive to the polarity and pH, and reasonably stable to physiological conditions. Porphyrin was used as the energy acceptor because its Q-band absorption overlaps BODIPY’s emission. Although this system is built by noncovalent approach, its accuracy of controlling location and orientation can compare well with that by the covalent approach because of the exceptionally strong binding ability, unambiguous binding stoichiometry, and geometry of permethyl-β-cyclodextin with porphyrin. In addition, the obtained assembly has benign water-solubility, allowing its potential application in biological system.

**Figure 9:**
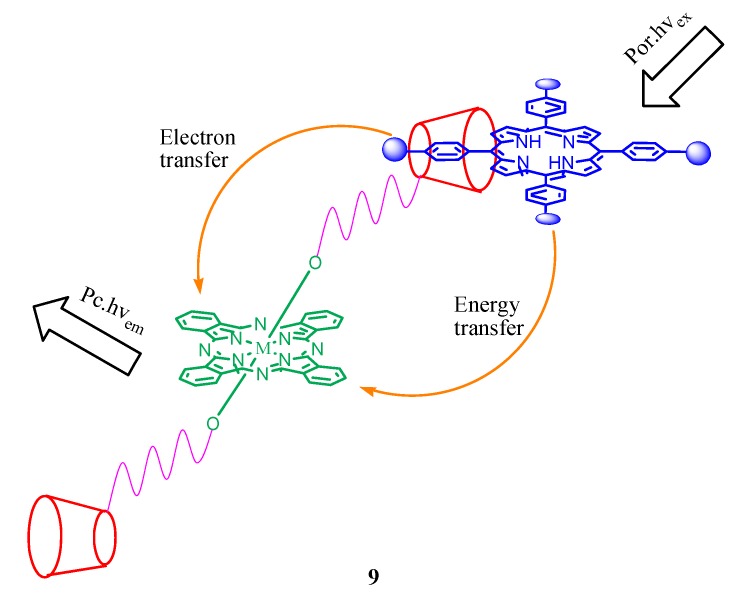
Energy- and electron-transfer between the porphyrin and pthalocyanine connected through a β-cyclodextrin spacer (**9**).

**Figure 10:**
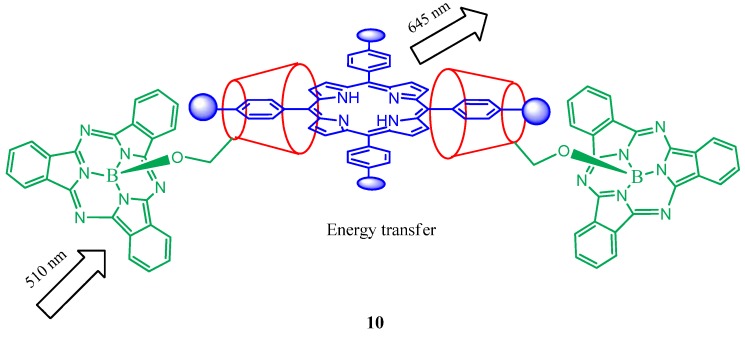
Representation of the 2:1 host-guest complex (**10**) formed by a β-cyclodextrin-conjugated subphthalocyanine and tetrasulfonated porphyrin and its energy transfer abilities.

A series of host-guest 1:1 complexes **9** (Figure 9) formed between a tetrasulfonated porphyrin and several silicon (IV) phthalocyanines substituted axially with two permethylated β-cyclodextrin units via different spacers has been reported [50]. It is found that the two major photoinduced processes, namely fluorescence resonance energy transfer and charge transfer are involved between the porphyrin and pthalocyanine which are controlled by the spacer between the β-cyclodextrin units and the silicon centre of phthalocyanine. The spacer having a tetraethylene glycol or oxo linker exhibited an efficient charge transfer from the excited phthalocyanine to the porphyrin entity. A stable 2:1 host-guest complex (Figure 10) was also reported to form between a β-cyclodextrin-conjugated subphthalocyanine and a tetrasulfonated porphyrin in water [51]. The complex exhibits energy transfer from the excited subphthalocyanine to the porphyrin core with an excitation energy transfer quantum yield of 0.38.

A cytochrome P_450_ model comprised of a 1:1 inclusion complex [tetrakis(*p*-sulfonato-phenyl)porphyrinato]Fe(III), TPPSFeCl, TPPS4FeCl, and 2-hydroxypropyl-β-cyclodextrin, HP-β-CD is reported by Khavasi *et al*. [52]. The inclusion behavior of HP-β-CD depends on the pH and the inclusion ability of the TPPS4FeCl/HP-β-CD system is strongest in neutral solution. This inclusion complex has been examined for the aqueous catalytic oxidation of styrene under different reaction conditions and is observed that addition of HP-β-CD to the porphyrin solution increases catalytic activity and selectivity of the system. Tetrakis(permethyl-β-cyclodextrin)-modified Zn(II) porphyrin) and tetra(β-cyclodextrin)-modified Zn(II) porphyrin were synthesized via click chemistry by Liu *et al*. [53]. Intermolecular inclusion complexation of these structurally similar systems with tetrasodium tetraphenylporphyrin tetrasulfonate gave two distinctly different nano-architectures with alternate porphyrin and cyclodextrin arrays, which were proven to be network and nanorod aggregates, respectively. The authors also proposed the mechanism that results in nanorod to network aggregates transition observed in these nanosystems. Solvent controlled photoinduced electron transfer (PET) between an anionic porphyrin and a cyclodextrin modified carbon nanotube is also reported by the same group and is shown that the CD cavities play a vital role on the control of the PET process [54]. 1:1 inclusion complexes are reported to form from cationic 5,10,15,20-tetrakis[4-(3-pyridinium-propoxy)phenyl]porphyrintetrakis-bromide (TPPOC3P) and β-cyclodextrins [55] or from *meso*-tetrakis(2-hydroxy-5-sulfonatophenyl)porphyrin with six different cyclodexrtins [56]. Kano *et al*. reported a novel system in which an anionic cyclodextrin bonded electrostatically with cationic cytochrome c where the cyclodextrin covers the hemin pockets of about two-thirds of the cytochrome molecules. This association complex is then further complexes with a non-ionic porphyrin to form a ternary complex in which static fluorescence quenching of the porphyrin by cytochrome c takes place [57].

**Figure 11:**
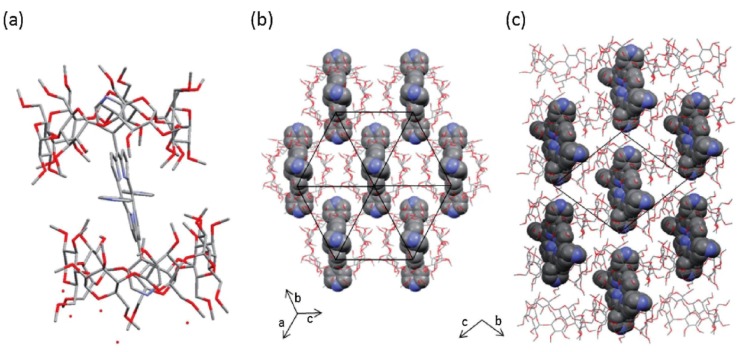
Crystal structure porphyrin-CD complex from ref [58]; (**a**) unit cell, (**b**) (111) plane and (**c**) along the axis. The hydrogen atoms are omitted (reproduced with permission from The Chemical Society of Japan).

A single crystal X-structure of porphyrin bicapped with cyclodextrin (Figure 11) was reported recently by Tsuchiya *et al*. [58]. 

In this study the authors used 2,3,6-Trimethyl-β-cyclodextrin and 5,10,15,20-tetrapyridylporphyrin to form host guest complex and got crystal from an aqueous solution of porphyrin containing an excess amount of cyclodextrin at 60 °C.

**Figure 12:**
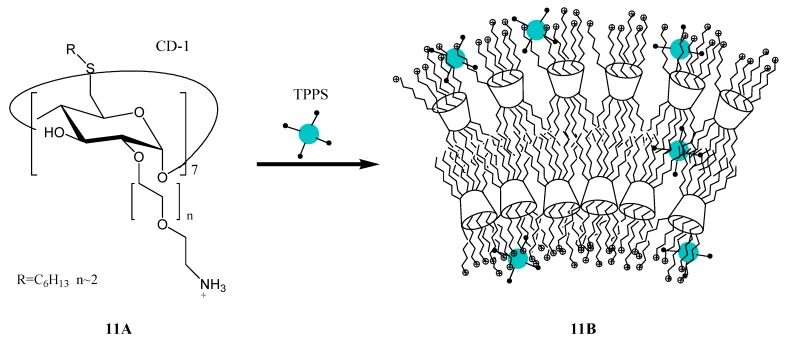
Formation of biocompatible nanoparticles (**11B**) of cationic amphiphilic cyclodextrins entangling the anionic porphyrins.

The capability of biocompatible nanoparticles (NPs, Figure 12, **11B**,) of cationic amphiphilic cyclodextrins (Figure 12, **11A**), entangling the anionic hydrophilic 5,10,15,20-tetrakis(4-sulfonatophenyl)-21*H*,23*H*-porphyrin (TPPS) to facilitate photoinduced energy and electron transfer with suitable donor and acceptor guest molecules has been demonstrated by Callari and coworkers [59]. Anthracene (AN) and anthraquinone-2-sulfonate (AQS), which could be employed as appropriate energy donor and electron acceptor, respectively are found to be trapped within this NPs network. Fluorescence experiments provided clear evidence that the amphiphilic NPs strongly encourage singlet-singlet energy transfer from AN to TPPS as well as photoinduced electron transfer from TPPS to AQS. In view of the biocompatibility and drug-carrying properties of the NPs used, these results, according to the authors, can be of interest from the perspective of designing photo-activated drug delivery systems.

**Figure 13:**
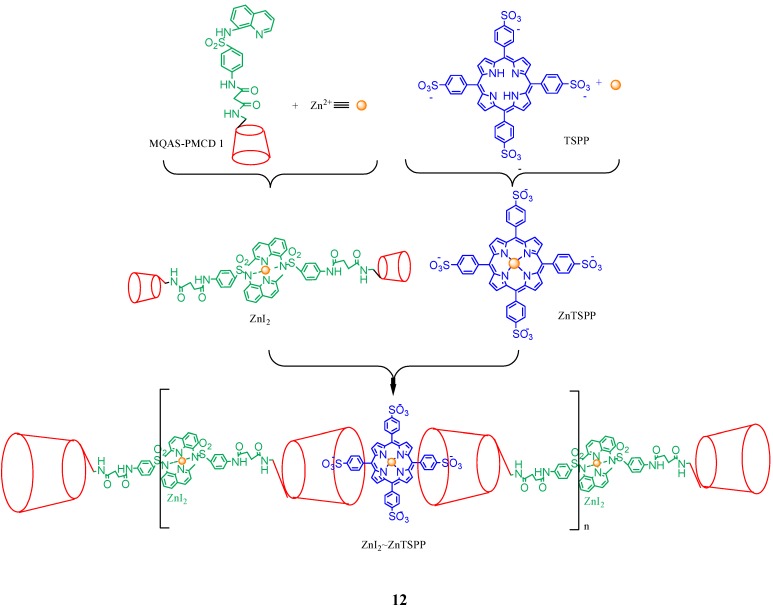
Schematic representation of the synthesis of the Zn^2+^/cyclodextrin/porphyrin triad (**12**).

An environment-sensitive Zn^2+^/cyclodextrin/porphyrin triad supramolecular assembly **12** (Figure 13) was constructed by Yu *et al*. [60]. When interacting with the cell membrane, this assembly was observed to be disrupted to release its porphyrin components. While the porphyrin part entered the cells, the other components of the complex remained in the cell membrane. The transmembrane dissociation property makes this supramolecular assembly an excellent candidate for the application in drug delivery.

The photoinduced energy and electron transfer within cyclodextrin-porphyrin system with or without suitable donor and acceptor guest molecules is investigated by many and these results can be of great interest from the perspective of designing photo-activated drug delivery systems and suitable PDT agents.

Chemical and biological nanocavities are provided to an anionic 5,10,15,20-tetrakis(4-sulfonatophenyl)-porphyrin (TPPS) via forming 1:1 and 1:2 encapsulation complexes with a quaternary ammonium modified β-cyclodextrin (QA-β-CD) and human serum albumin (HSA) protein in aqueous solution [61]. The effect of O_2_ on the relaxation of the triplet state of the free and encapsulated TSPP is studied and the obtained results could be correlated with the effect provided by those chemical and biological cavities. These nano-cavities have notable effects on the fluorescence lifetimes also. The observed difference, longer triplet lifetime upon encapsulation, according to the authors, is relevant to the efficiency of this porphyrin in photodynamic therapy. An emitting nanoassembly composed of a novel amphiphilic cyclodextrin functionalized with a covalently appended fluorophore and an anionic porphyrin internalizes effectively in tumor cells, allowing simultaneously the detection of carrier and photosensitizer has also been reported recently [62].

The combination of monocationic *meso*-substituted porphyrin 5-[4-(1-dodecanoylpyridinium)]-10,15,20-triphenylporphine (TDPyP) with supramolecular aggregates of cationic amphiphilic β-cyclodextrin (SC6NH2) can act as singlet oxygen generation system having very high quantum yield (ΦΔ = 0.90). Although the yield of ^1^O_2_ generation was comparable to that obtained after TDPyP incorporation into cationic unilamellar liposomes of *N*-[1-(2,3-dioleoyloxy)propyl]-*N*,*N*,*N*-trimethylammonium chloride (DOTAP) , SC6NH2-bound TDPyP was more active than DOTAP-bound TDPyP in photosensitizing the inactivation of the Gram-positive methicillin-resistant bacterium *Staphylococcus aureus* (MRSA) [63]. In addition, the photo activated SC6NH2-bound TDPyP is also efficient in photokilling of Gram-negative bacterial pathogens, such as *Escherichia coli*. Transmission electron microscopy studies revealed that the high photobactericidal effect of the SC6NH2-bound TDPyP is the consequence of the carrier’s ability to facilitate an efficient crossing of the very tightly organized three-dimensional bacterial outer wall by the embedded porphyrin so that a prompt interaction between the short-lived photogenerated ^1^O_2_ and the targets tissues can take place. The photodynamic activity of a novel porphyrin derivative associated with β-cyclodextrin is also reported by Mora *et al*. [64].

**Figure 14:**
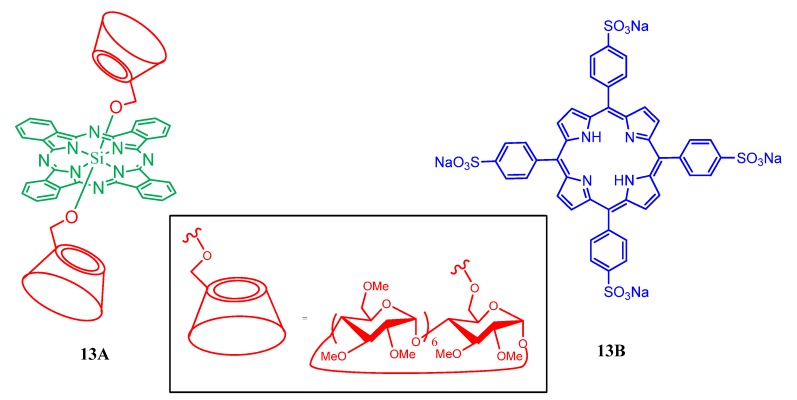
Si(IV) phthalocyanine conjugated permethylated β-cyclodextrin (**13A**) and tetrasulfonated porphyrin (**13B**) used to make host-guest complex.

The linear nano-architecture of an inclusion complex of 5,10,15,20-tetrakis(4-sulfonatophenyl)-porphyrin with mono-6-deoxy-6-iodo-β-cyclodextrin having a 6,7-bis(methylsulfanyl)-2,3-bis(2-cycanoethylsulfanyl)tetrathiafulvalene linker is reported by Zhang *et al*. [65]. Upon light irradiation, efficient quenching of the porphyrin fluorescence observed in this supramolecular complex, which is due to the significant photoinduced electron transfer (PET) process between the tetrathiafulvalene moiety and the porphyrin unit. The porphyrin fluorescence is then recovered by the formation of tetrathiafulvalene cations in the presence of H_2_O_2_. The same research team also reported linear supramolecular architectures with a width of about 2 nm and a length ranging from hundreds of nanometers to micron dimension were conveniently constructed by interacting 5,10,15,20-tetrakis(4-sulfonato-phenyl)porphyrin or its Zn(II) derivative with bis{4-methyl[1–3]triazolyl}malonic ester-bridged bis(permethyl-β-cyclodextrin) having an attached fullerene. Efficient photoinduced electron-transfer between porphyrin and C_60_ moieties are observed in these new fullerene bridged bis(permethyl-β-cyclodextrin)-porphyrin [66]. Leng *et al*. reported a stable 1:1 host-guest complex in which a Si(IV) phthalocyanine conjugated axially with two permethylated β-cyclodextrin units (Figure 14, compound **13A**) and a tetrasulfonated porphyrin **13B** (Figure 14). This complex exhibited light-harvesting property and worked as an efficient photosensitizing system, causing photokilling of HT29 human colon adenocarcinoma cells efficiently [67].

Densely packed hybrid monolayers of amphiphilic cyclodextrins incorporating hydrophilic porphyrins were prepared at the air/water interface through electrostatic interaction by Valli *et al*. and are transferred onto quartz substrates by Langmuir-Schaefer deposition [68]. The resulting multilayers exhibit a good response to light excitation as proven by fluorescence emission, triplet-triplet absorption and singlet oxygen photogeneration. A supramolecular self-assembly constructed by perylene-bridged bis(β-cyclodextrin)s with water-soluble porphyrin through hydrophobic interactions is also reported recently in which a strong excitonic coupling interactions between perylene backbones and included porphyrins is observed [69].

The interactions of tetraphenylporphyrin (TPP), [tetraphenylporphyrinato]Co(II) (CoTPP) and a protein in the presence of a cyclodextrin derivative, heptakis(2,6-di-*O*-*n*-octyl)-β-cyclodextrin (Oc-β-CD), have been investigated by Liping *et al*. [70]. In the presence of Oc-β-CD, significant increase of TPP fluorescence was observed, but the increased fluorescence was quenched by the presence of CoTPP. Again the quenching fluorescence of TPP was restored upon interacting with protein. The restoration of TPP fluorescence is fast and accomplished upon interaction with bovine serum albumin (BSA) or human serum albumin (HSA). The mechanism of TPP fluorescence quenching is proposed to be the formation of a ground-state complex of TPP and CoTPP, and the fluorescence restoration is attributed to the binding of CoTPP with the protein molecule which destroys the aggregate, releasing the free base porphyrin. According to the authors this method could be used for the direct assay of HSA content in human serum.

Investigation on cyclodextrin-porphyrin systems have led to a significant expansion of the field of artificial enzymes or enzyme mimics. It is obvious that many novel catalysts can be constructed, based on cyclodextrins scaffold and attachment of catalytic active porphyrin function to a cyclodextrin can afford interesting enzyme mimics.

A new type of artificial smart bifunctional enzyme with both superoxide dismutase (SOD) and glutathione peroxidase (GPx) activities was successfully constructed by the self-assembly of [tetra(1-(1-adamantylmethylketone)-4-pyridyl)porphyrinato]Mn(III), (MnTPyP-M-Ad), with four suspensory adamantyl moieties and β-cyclodextrin-terminated temperature-sensitive copolymer through host-guest interaction in aqueous solution [71]. The Mn(III) porphyrin was designed as both a “supramolecular linker” and an efficient active site of SOD. This bifunctional enzyme exhibited stable SOD-like activity and high GPx catalytic efficiency with temperature responsive dependence characteristic. Another supramolecular artificial enzyme was successfully constructed by the self-assembly of the same Mn(III)*meso*-tetra[1-(1-adamantylmethylketone)-4-pyridyl]porphyrin (MnTPyP-M-Ad) and cyclodextrin-based telluronic acid (2-CD-TeO_3_H) through host-guest interaction in aqueous solution. The self-assembly of the adamantyl moieties of Mn(III) porphyrin and the β-CD cavities was also demonstrated along with this work. In this enzyme model, the Mn(III) porphyrin center acted as an efficient active site of SOD and tellurol moiety endowed GPx activity. The SOD-like activity of the new catalyst was found to be exhibit 2.56% of the activity of the native SOD. Besides this, enzyme model also showed a high GPx activity, and a remarkable rate enhancement of 27-fold compared to the well-known GPx mimic ebselen was observed. More importantly, this supramolecular artificial enzyme showed good thermal stability too [72].

**Figure 15:**
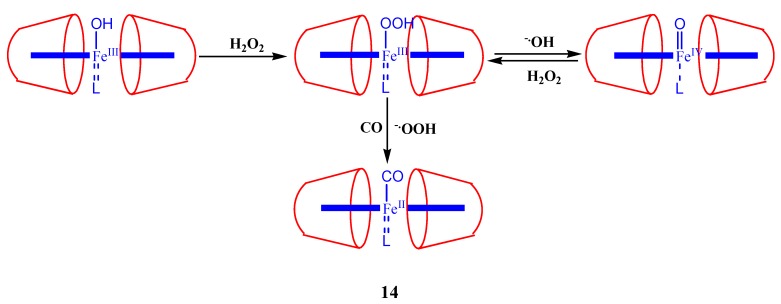
Schematic representation of iron-oxo intermediate formation after the uptake of H_2_O_2_ by a pyridine-coordinated ferric porphyrin encapsulated in the cyclodextrin dimer.

The synthesis, spectroscopic characterization and enzyme-like activities of manganese(III) complexes of 5[4-(6-*O*-β-cyclodextrin)-phenyl],10,15,20-tri(4-hydroxyphenyl)-porphyrin is reported by Oliveri *et al*. [73]. Stable J-aggregates of diprotonated 5,10,15,20-tetrakis(4-sulfonatopheny)porphyrin (H_4_TPPS^2−^) containing per-*O*-methylated β-cyclodextrin is prepared by Kano *et al*. after binding them to the surface of α-chymotrypsin (ChT) [74]. This porphyrin aggregates can function effectively to control/modulate the enzyme activity of chymotrypsin. The same group also successfully demonstrated how to to regulate α-chymotrypsin catalysis by iron porphyrins when a suitable cyclodextrin is introduced in the system [75]. The developments in enzyme mimic research carried out on porphyrin-cyclodextrin systems are so fast that in near future we can expect interesting artificial enzymes based on this combination. The uptake of H_2_O_2_ by a pyridine-coordinated ferric porphyrin encapsulated in a cyclodextrin dimer yielded variety of iron-oxo intermediates, as shown in Figure 15 [76].

There are also reports describing the binding of dioxygen, carbon monoxide or cyanide to the porphyrins associated with cyclodextrin analogy to the natural heme systems. In these host-guest inclusion model, cyclodextrin being regarded as the protein component, which acts as a carrier enveloping the active site of heme prosthetic group within its hydrophobic environment, provides a protective sheath for the porphyrin. Metalloporphyrins, on the other hand, could serve as the heme analogs in such heme protein model compounds [77,78,79,80,81].

Poly(acrylic acid) modified by supramolecular complex composed of 10,15,20-tris(4-sulfonato-phenyl)porphyrinato]Fe(III) and a per-*O*-methylated β-cyclodextrin dimer is reported recently. The oxygen uptake capability and subsequent autoxidation of the oxo-adduct of different combinations of these systems are then investigated in detail. (CD_A12). Kano *et al*. reported a 1:1 complex (hemoCD) of [5,10,15,20-tetrakis(4-sulfonatophenyl)porphyrinato]Fe(II) (Fe[II]TPPS) and a per-*O*-methylated β-cyclodextrin dimer having a pyridine linker (Py3CD). This hemo-CD binds O_2_ reversibly in aqueous solution. Encapsulation of the iron center of Fe(II)TPPS by two cyclodextrin truncated cones is supposed to be the determining factor for binding of O_2_ to the ferrous center of the porphyrin [79]. An anionic iron porphyrin complexed with per-*O*-methylated β-cyclodextrin dimer having an imidazole linker is reported to be a much better cyanide receptor *in vivo* than hydroxocobalamin [80].

## 3. Calixarene-Porphyrin Assemblies

**Figure 16:**
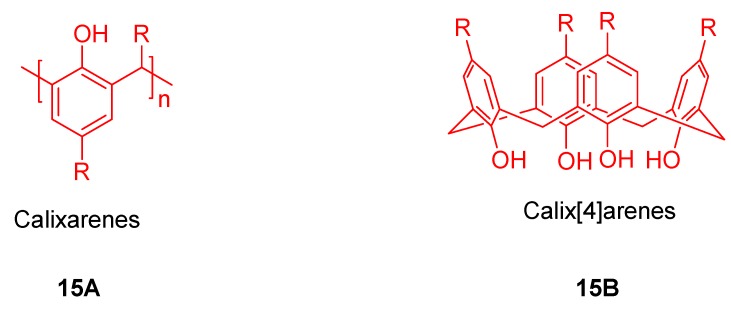
Schematic representation of calix[n]arene (**15A**) and calix[4]arene (**15B**).

The calixarenes are a class of cyclooligomers formed via a phenol-aldehyde condensation (Figure 16, compounds **15A**,**B**). They exist in a cup-like shape with a defined upper and lower rim and a centre annulus. Calixarenes have come a long way in offering tailor made candidates for diverse applications in the field of host-guest chemistry [25,26,27,28,29,82,83,84,29,82]. Calixarenes have many conformational isomers because of two possible rotational modes of phenol units. The size and geometry of calixarenes were determined by the functionalization of the upper rim, lower rim or both. Calixarenes are superior to crown ethers and cyclodextrins in the aspect. Interesting building blocks in supramolecular chemistry have been constructed by combining porphyrin and calixarene. These blocks possess different topologies and electronic properties, and their combination allows the creation of receptors taking advantage of both precursors.

Investigations on molecular tweezers formed by bridging of a calixarene fragment between two porphyrin rings attract a strong interest by many researchers [85,86,87,88,89,90,91,92,93,94,95,96,97]. A combination of these structural fragments in a single molecule via covalent bonds opens unique possibilities for building up cavities to bind both ionic and neutral species because the flexible porphyrinic ring can evolve toward a conformation that allows the complexation.

**Figure 17:**
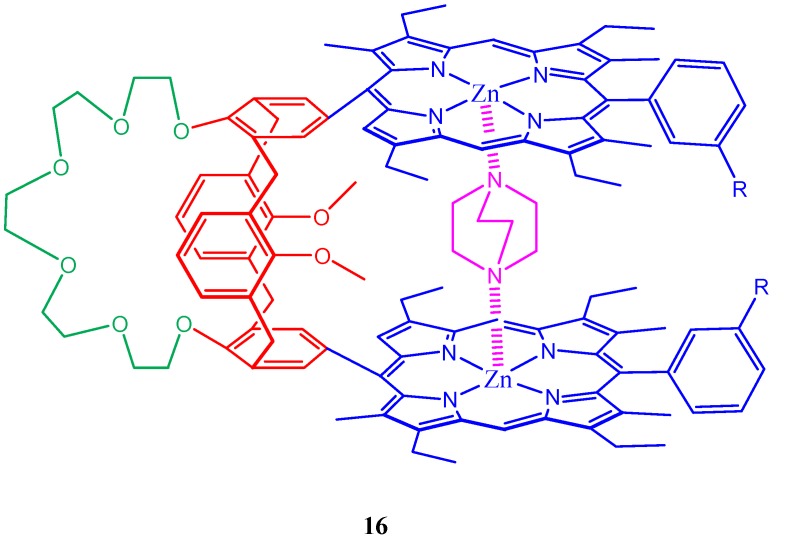
The heterotopic receptors based on bisporphyrin-calix[4]arene crown ether.

Synthesis of the heterotopic receptors on the basis bisporphyrin-calix[4]arene crown ethers was reported by Mamardashvili et al. [86]. The idea is to covalently combine in one molecule a crown-calix[4]arene fragment and two appropriately functionalized porphyrin macrocycles (Figure 17). This system allows the creation of sterically preorganized hetrotropic receptors possessing polyfunctional complexing power toward both charged particles and neutral molecules of various natures. Their spectral properties and complexing ability toward the alkali metal cations, organic diamines, and dicarboxylic acid esters were also described. A 1,3-bis[Zn(II) porphyrin]-substituted calix[4]arene (cone conformation) is reported by Arimura et al. which served as selective molecular tweezers for DABCO, leading to the formation of calix[4]arene-supported porphyrin-Zn-DABCO-Zn-porphyrin ensemble [87]. ^13^C-NMR longitudinal relaxation time studies of these molecular tweezers upon DABCO incorporation is also discussed in the same paper.

**Figure 18:**
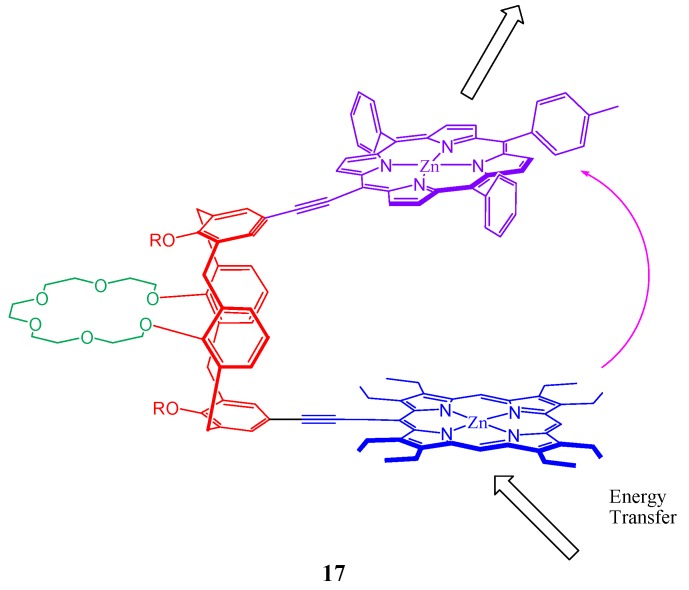
Possible photoinduced energy transfer observed in the tweezer molecular system (**17**) containing porphyrins, calixerene and crown ether.

Synthesis of a Pacman heterobis(porphyrin) containing an [octaethylporphyrinato]zinc (ZnOEP) energy donor and a (triarylporphyrinato)zinc (ZntPP) energy acceptor around a crown[6]-dipropoxy-calix[4]arene spacer is described (Figure 18). Remarkable photoinduced energy transfer is found to be observed in this tweezer molecular system [88]. This was evidenced by increase of luminescence in the ZntPP moiety and a corresponding quenching of the OEP moiety’s emission, which are independent of the excitation wavelength. Ivanova *et al*. showed that the distance between the reaction centers of the porphyrin fragments of their calixarene-bis-porphyrin system can be controlled by varying the solution acidity and thus arousing the possibility of developing molecular switches [89]. A Zinc complex of *meso*-(aminophenyl)-substituted calixarene-bis-porphyrin was also reported which has been found to form a stable 1:1 complex with dimethyl maleate in toluene [90]. Induction of chirality on noncovalently bound-[Ru(phen)_3_]^2+^ enantiomers in a porphyrin-(bis)calixarene assemblies is demonstrated by D’Urso *et al*. [91].

It has been observed that several calixarene-porphyrin-tweezer systems are capable of size-selective complexation with fullerenes. Designing effective host molecules for such fullerene selectivity is highly important to reduce costs due to laborious fullerenes purification and its isolation procedures and thus to facilitate their application in chemistry and material science. Also porphyrin-fullerene architectures can lead to molecular assemblies ideally suited for devising integrated, multicomponent model systems to transmit and process solar energy [98,99]. The geometrical complimentary of concave calixarene cavities and spherical fullerenes has attained active interest of chemists. At the same time the curved π surface of C60 or C70 fullerenes can interect with the metalloporphyrin moiety by attractive π-π interactions, which can be applied to produce discrete host-guest systems possessing unique properties based on diverse supramolecular charge transfer.

**Figure 19:**
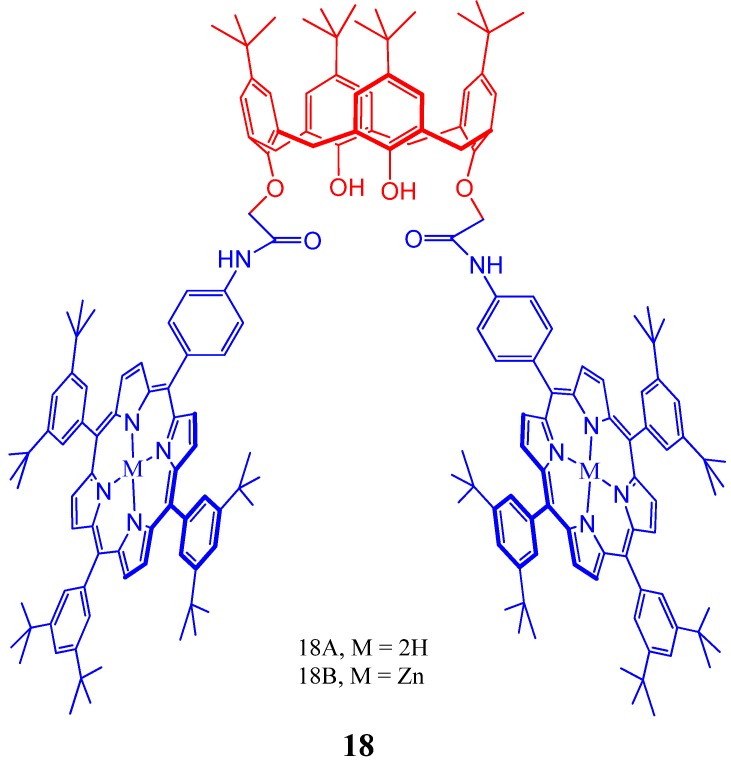
Calixarene-bisporphyrins system (**18**) capable of achieving electronic charge transfer during host-gust interactions.

**Figure 20:**
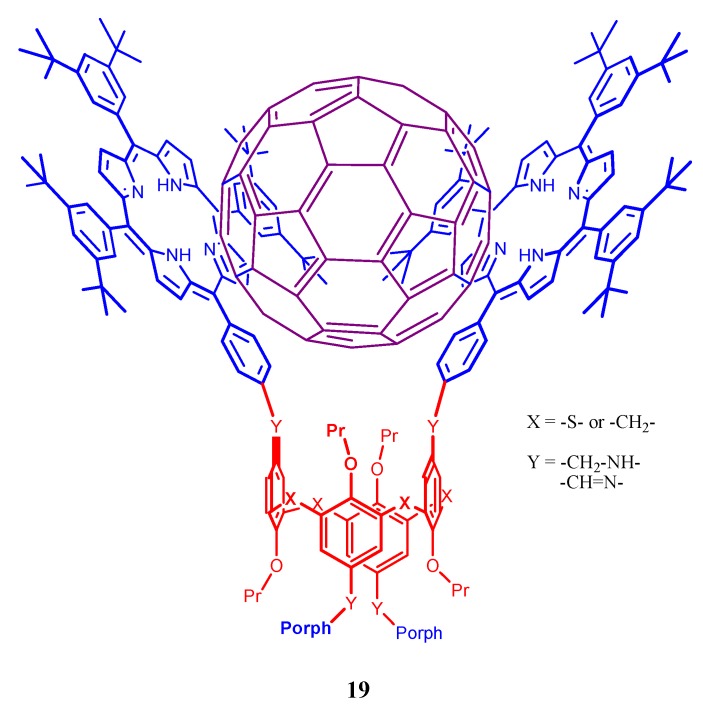
Molecular tweezer (**19**) based on aminoporphyrin and tetraformylcalixarene and its selectivity towards C_70_.

Supramolecular interactions of calixarene scaffold bearing bisporphyrins as hosts with fullerenes have been reported by many [86,95,96,97]. Grimm *et al*. reported an oxidative charge transfer (*i.e.*, electron transfer from the bisporphyrin to the fullerene) for the C_60_ inclusion complexes, while a reductive charge transfer (*i.e.*, electron transfer from the fullerene to porphyrin) is operative in the endohedral metallofullerene host-guest complexes, (Figure 19) [95]. Molecular tweezer **19** (Figure 20) based on aminoporphyrin and tetraformylcalixarene was also reported to form 1:1 complexes with C_60_ and C_70_ fullerenes and possess high selectivity toward C70 [96].

**Figure 21:**
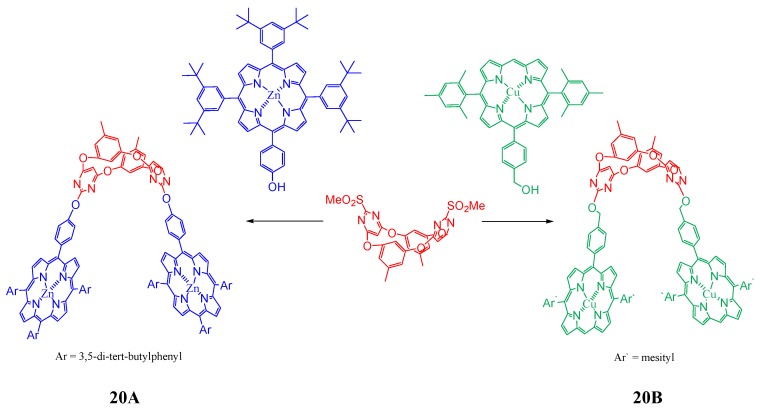
Oxaclixarene tweezers based on porphyrin (**20A**) and corrole (**20B**).

An oxacalix[2]arene[2]pyrimidine-bis(Zn-porphyrin) tweezer **20A** (Figure 21) was demonstrated to be an excellent selective receptor towards fullerene C_70_ by Rossom *et al*. [97]. This tweezer is found to form 1:1 complex with C_70_ while no complexation occurred with C_60_. On the other hand an analogous oxacalix[4]arene-bis(Cu-corrole) **20B** (Figure 21) conjugate did not show any measurable C_60_ or C_70_ fullerene binding.

**Figure 22:**
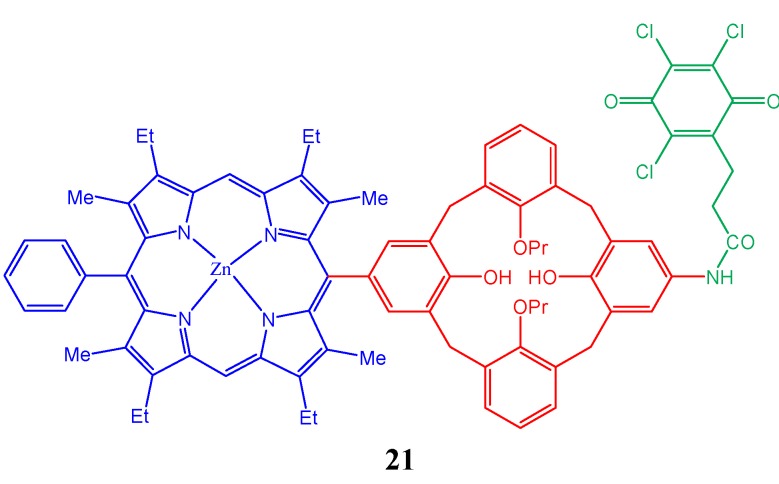
Schematic representation of the supramolecular system (**21**) based on porphyrin-calixarene- trichloroquinone.

Conjugation/coordination of a third functional substrate to the porphyrin-calixarene system also allows construction of devices such as donor-acceptor systems for photoinduced electron transfer. A new calixarene-based supramolecular system, **21** (Figure 22), containing porphyrin and trichloroquinone is also reported in which through-space donor-to-acceptor electron transfer is observed upon photoexcitation [100]. Synthesis of mono- and heteronuclear lanthanide-containing complexes on the basis of 1,3-disubstituted *p*-*tert*-butylcalix[4]arenes modified with porphyrin and benzimidazole fragments are reported recently [101]. The analysis of the relationship between spectral-luminescent properties of these compounds and their structure has also been carried out. A calixarene-based bisporphyrin in which the calixarene scaffold is connected with porphyrin units by an alkyl chain as a spacer is reported and it exhibits extremely high affinity for diazabicyclo-[2.2.2]octane (DABCO) [102].

**Figure 23:**
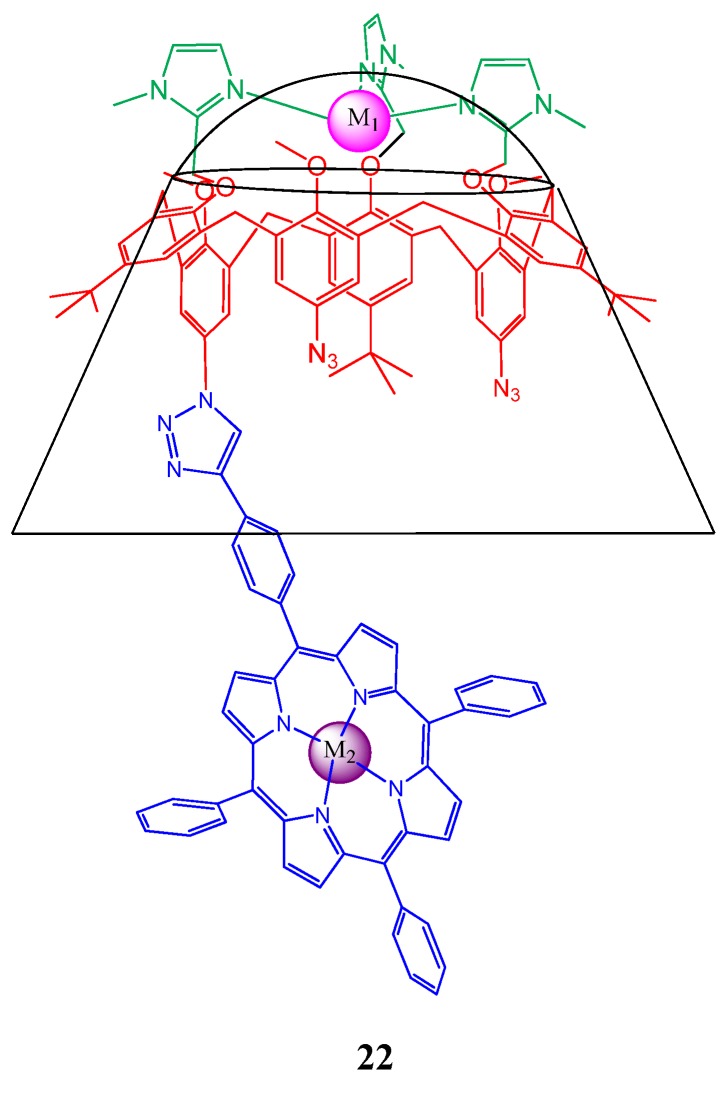
Calix[6]arene based ditopic ligand (**22**) bearing a tridentate imidazole metal coordination site at its small rim and a single porphyrin ligand appended to its large rim.

Monnereau *et al*. synthesized a novel calix[6]arene derivative **22** (Figure 23) bearing a tridentate imidazole metal coordination site at its small rim and a single porphyrin ligand appended to its large rim [103]. This calixarene derivative behaves as a ditopic ligand because in addition to the metallatedporphyrin, the tris-imidazole site are readily accommodate one Zn(II) cation.

The coordination of Zn to the tris-imidazole is found to rigidify the calixarene, locking it into a cone conformation, and concomitantly provides a new coordination site controlled by the calixarene cavity. The corresponding calix(Zn)P(Zn) is then able to accommodate 1 equivalent of primary amine inside the calixarene cavity and is the thermodynamically favored one too. The use of a diamine with a C-12 aliphatic chain led to the formation of a heterodipotic complex highlights the possibility of generating cooperative interactions between the three components; calixarene-guest-porphyrin similar to interactions governing enzymatic catalysis. Synthesis and conformational analysis of some porphyrin derivatives substituted with calyx[4]arene subunits were also carried out recently by Holler *et al*. [104].

**Figure 24:**
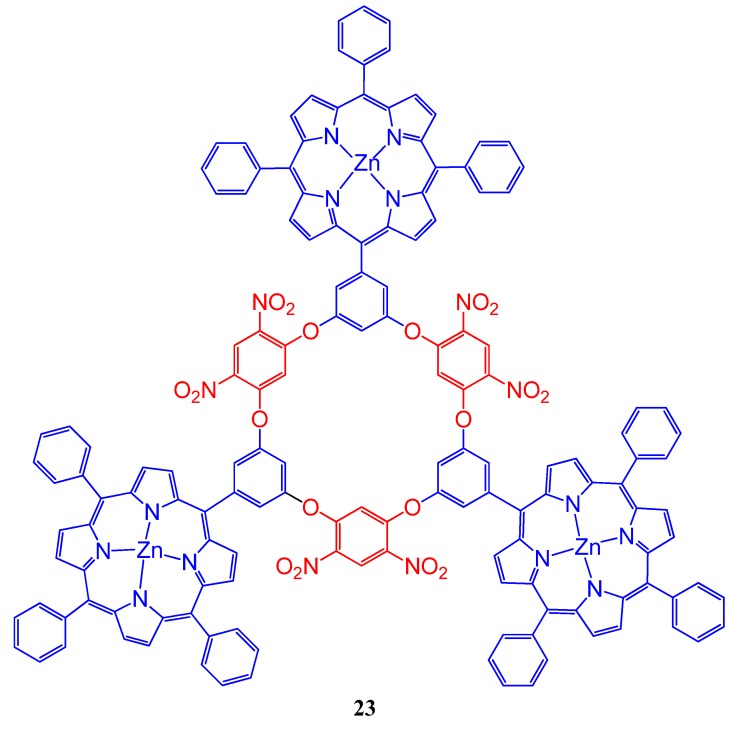
The fluorogenic oxacalix[6]arene system (**23**) bearing three Zn(II) porphyrins.

**Figure 25:**
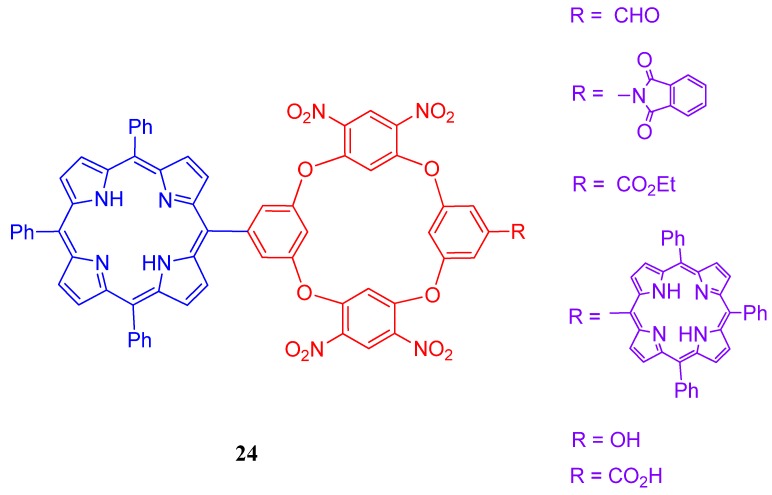
Schematic representation of functionalized oxacalix[4]arene porphyrins, **24**.

Wu and coworkers synthesized a new fluorogenic oxacalix[6]arene system **23** (Figure 24) bearing three Zn(II) porphyrins. This trimeric system and crystal violet found to form 1:1 complex and the fluorescence intensity of the porphyrin system is quenched regularly due to this complex formation. According to the authors, this compound can be found an application in the fluorometric titration method as an efficient detection agent for crystal violet with good linearity in the concentration range of 1.40–37.4 μmol/L [105]. Some functionalized oxacalix[4]areneporphyrins, **24**, (Figure 25) (R = CHO, CO_2_H, CO_2_Et, OH, phthalimido), were synthesized via a high-yielding “3+1” condensation between *meso*-(3,5-dihydroxyphenyl)triphenylporphyrin and readily available new fluorodinitrobenzene-containing trimers were synthesized and photophysical properties were discussed by Jiao *et al*. [106].

**Figure 26:**
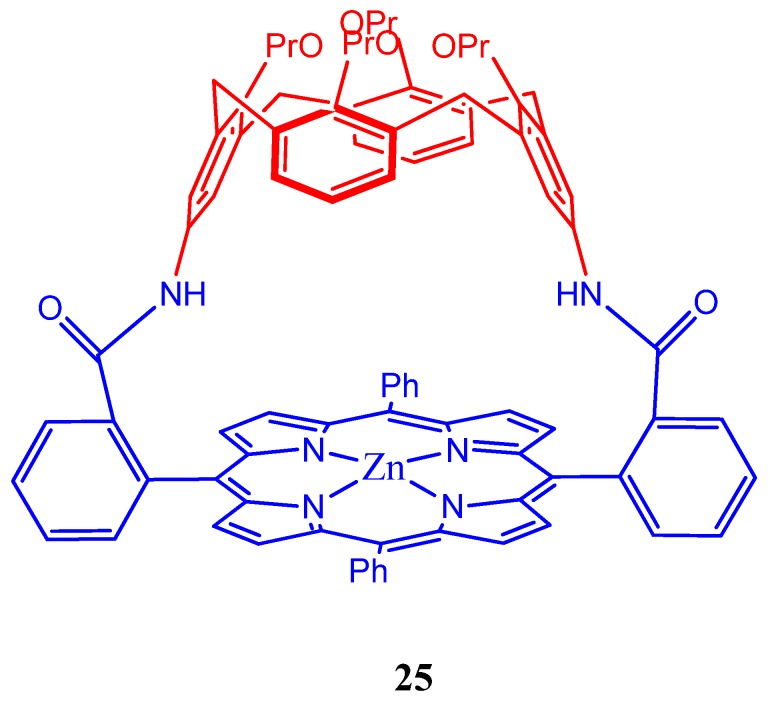
Calix[4]arene-capped Zn(II) porphyrin (**25**) capable of recognizing *N*-containing aromatic species.

Highly shape selective guest encapsulation is achieved recently by a calix[4]arene-capped Zn(II) porphyrin **25** (Figure 26) which precisely recognizes the shape of small N-containing aromatic species in the confined inner cavity [107].

Construction of multilayers comprising calixarene and porphyrin units is another important research area [108,109,110,111,112,113,114] and a promising application of such porphyrin based receptor arrays is in the field of cross responsive sensors for the generalized detection of volatile organic compounds and for recognition of organic compounds. For example, 4 zinc porphyrin molecules were linked covalently to a central amphiphilic calix[4]arene molecule to form a zinc calix-porphyrin, which displays enhanced sensitivity to dibutylamine compared to both the discrete zinc porphyrin and two-component mixtures of zinc porphyrin with calixarene [109]. The dense packing of the monomeric porphyrin moieties leads to a high absorbance per layer and thus a large sensing signal compared to other LB films capable of amine-detection. This zinc calix-porphyrin is selectively responsive to the secondary amine, dibutylamine, compared to the primary and tertiary compounds. Mixed LB Films containing a cationic porphyrin and an anionic calix[8]arene octacarboxylic acid derivative were prepared and analyzed [110]. The porphyrin aggregation is a result of a double comb configuration, where porphyrins from opposite layers are interwoven in a linear infinite J-aggregate. The cationic porphyrin tends to self-aggregate strongly, provided the electrostatic repulsions of their peripheral groups are cancelled by the anionic groups of the calixarene matrix.

**Figure 27:**
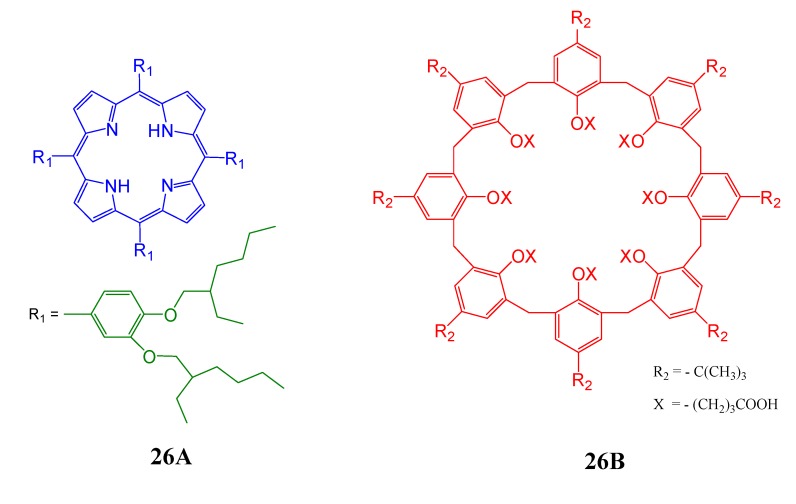
Schematic representation of 5,10,15,20-tetrakis[3,4-bis(2-ethylhexyloxy)phenyl]-21*H*,23*H*-porphine (**26A**) and *p*-*tert*-butylcalix[8]arene (**26B**).

The gas sensing capabilities of Langmuir-Blodgett (LB) mixed films of 5,10,15,20-tetrakis[3,4-bis(2-ethylhexyloxy)phenyl]-21*H*,23*H*-porphine (Figure 27, **26A**) and *p*-*tert*-butylcalix[8]arene (Figure 27, **26B**) have been studied by Roales *et al*. [111]. Although the parent is known to be very sensitive to NO_2_ gas, this study demonstrated that the LB layer matrix was found to improve the sensing properties of the porphyrin molecule in the solid state. In AFM images, this LB films appeared with sharper surfaces than those made of pure porphyrin, allowing a better accessibility of the gas molecules to the active binding sites. The same porphyrin was also highly sensitive to low concentration of NO_2_ in nitrogen when organized at the air-water interface using the host calix[8]arene matrix mentioned above [112]. The distribution of the porphyrin molecules at the air-water interface in the calixarene matrix was found to be more homogeneous than that of the pure porphyrin monolayers. According to the authors, this homogeneous organization of porphyrin molecules in these mixed films is clearly manifested by the enhancement of response (faster), reproducibility (higher), sensitivity (higher) and temperature (wide range, being interesting for industrial applications) during exposure to NO_2_, in comparison with those results previously obtained for the pure porphyrin film.

Zn and Mn derivatives of 5,10,15,20-tetrakis[3,4-bis(2-ethylhexyloxy)phenyl]porphyrins (EHO) have been mixed with calix[8]arene to enhance to the quality of the porphyrin films and their response to amine gases and also to increase the film lifetime [113]. ZnEHO showed considerable spectral changes under exposure to each of the primary, secondary and tertary amine gases when compared to that of MnEHO and the authors claimed that ZnEHO is highly efficient for amine sensing in a sensing device.

The self-assembly of charged, water soluble porphyrins as well as calixarenes also provide important host-guest systems for variety of applications [115,116,117,118,119,120,121,122,123]. D’Urso *et al*. employed a water-soluble calixarene (1,6-bis[5,11,17,23-tetrakis(trimethylammoniomethyl)-25,26,27-tripropoxy-28-(oxy)calix[4]arene]) as a templating agent for the assembly of a tetraanionic porphyrin ([tetrakis(4-sulfonatephenyl)porphyrinato]Cu(II)) [115]. The self-assembly in this condition resulted the noncovalent synthesis of 2-dimensional or 3-dimensional multiporphyrin assemblies and remarkable stability of these assemblies permitted a stepwise synthesis that makes it possible to choose the desired porphyrin sequence.

Noncovalent synthesis of discrete supramolecular entities was also reported by Tabbi *et al*. [116]. In this work, metalated *meso*-tetrakis(*N*-methyl-4-pyridyl)porphyrin (MTMPyP) and 5,11,17,23-tetra-sulfonato-25,26,27,28-tetrakis-(hydroxylcarbonylmethoxy)-calix[4]arene (C4TsTc) were used as key components for building up discrete supramolecular entities starting from the formation of the template species MTMPyP:C4TsTc (1:4, M = Cu, Zn). The stepwise addition of further amount of porphyrin allows the facile noncovalent synthesis of discrete supramolecular systems (2:4 and 3:4) which can be built up just by programming the right stoichiometric addition of the proper porphyrin. The authors reported that the electrochemistry of each of these supramolecular complexes is different from that of the parent components. This anomalous behavior can be explained only considering each of these supramolecular complexes as a unique entity, in which internal electronic communication might occur. A highly nanoporous material has been obtained by self-assembly of the polyanionic calixarene 5,11,17,23-tetrasulfonato-25,26,27,28-tetrakis(hydroxycarbonylmethoxy)calix[4]arene (C4TsTcn-) in the cone conformation and the tetracationic *meso*-tetrakis(4-*N*-methylpyridyl)porphyrin (H2TMPyP) building blocks [117,118]. This supramolecular zeolite-like structure was successively functionalized by diffusion and coordination of metal ions to form a new bi-functionalized nanoporous material containing a porphyrinic pigment together with a metal center. Supramolecular tetramer assemblies with parallel and perpendicular arrangement of tetrapyrrole macrocyclic rings are synthesized from calix[4]arene-bis(tin(IV)porphyrin systems by Mamardashvili *et al*. [119]. Anionic bis(*p*-sulfonatocalix[5]arenes) and cationic porphyrins, respectively, are self-assembled to form two well-defined nano-architectures with 2-D netlike and 1-D linear topological structures, in which an unambiguous PET process is observed in nanoscale region [120].

## 4. Resorcinarenes-Porphyrin Assemblies

**Figure 28:**
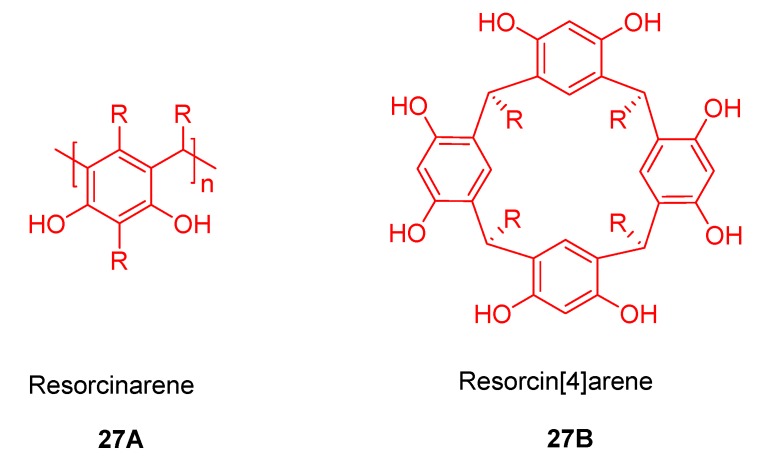
Schematic representation of resorcin[n]arene (**27A**) and resorcin[4]arene (**27B**).

Resorcinarenes, which are cyclic tetramers of resorcinol **27A**,**B** (Figure 28), are valuable three dimensional building blocks for molecular recognition. Usually in resorcinarenes, the resorcinol rings are connected with bridging moieties which results in a locked conformation called a cavitant. They are excellent molecular receptors that contain an enforced cavity large enough to accommodate simple organic compounds or ions [124,125,126,127,128]. Resorcinarenes can be functionalized by introducing many different ligand moieties containing atoms O, S, N, and P both at upper and lower rim.

Resorcinarenes are reported to form host-guest complexes with a variety of guest molecules, mostly with small organic molecules and ions, but also with alkali metal cations. Introduction of electronically and sterically diverse groups on the hydroxyls (upper rim) of resorcinarene significantly alters their physical, conformational, and binding properties. Also the hydroxyl groups at the upper rim of resorcinarene, or attached substituents, can engage in hydrogen bonding, while the π-basic cavity can form cation-π, CH-π, and π-π interactions with the guest. Resorcinarenes can also be employed as building blocks for self-assembled mono- and multilayers and can act as precursors to carceplexes, carcerands or hermicarcerands. All these fascinating properties of resorcinarenes make this class of compounds very prospective from the point of view of supramolecular chemistry, creation of valuable receptor molecules as well as new systems for biomimicking. Surprisingly, combination of porphyrin-resorcinarene systems are not much studied when compared to the corresponding porphyrin-calixarene or porphyrin-cyclodextrin conjugates.

**Figure 29:**
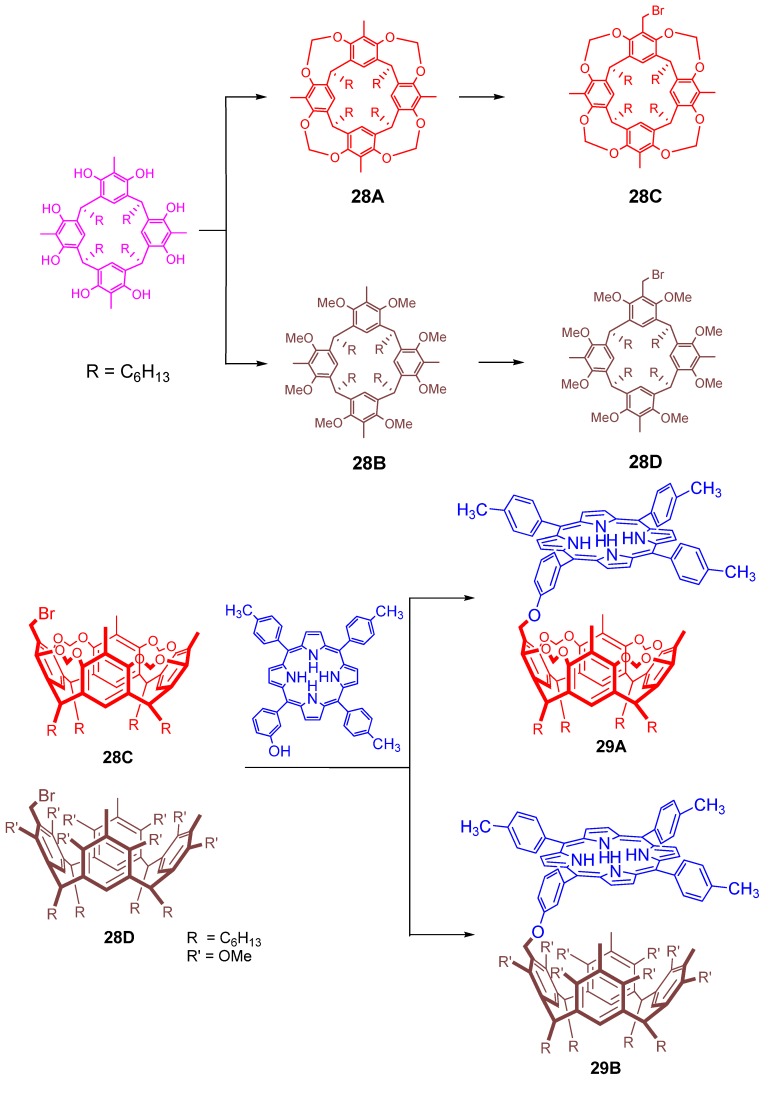
Synthetic pathways of resorcin[4]arene (**28A-D**) for the preparation of porphyrin conjugated rigid resorcin[4]arene (**29A**) and its flexible analogue (**29B**).

The present authors are interested in the synthesis of covalently bonded porphyrin-resorcinarene conjugates [129]. The strategy we employed was to construct functionalized resorcinarene moieties and to couple them with suitable porphyrins. The resorcinarene framework used is either conformationally rigid cavitand or flexible unfirm structure in order to appropriately tune the flexibility and rigidity of this host receptor for the encapsulation characteristics. The conformationally locked bowl shape resorcin[4]arene (Figure 29, **28A**), was synthesized by reacting parent octahydroxyresorcinol with BrClCH_2_, which introduced methylene bridges between four sets of proximate oxygens of resorcin[4]arenes. The synthesis of the unlocked compound **28B** (Figure 29) was achieved by the reaction of octa-hydroxy resorcinol with methyl iodide [129,130]. Monobromination of one of the benzylic hydrogens of both resorcin[4]arenes upper rim is then carried out for introducing a suitable functional group to couple with porphyrins. The monobromomethylresorcin[4]arenes **28C,D** (Figure 29) were finally reacted with meso-5-(3-hydroxyphenyl)-10,15,20-tritolulyporphyrin to obtain the targeted cavient as well as the flexible unfirm resorcin[4]areneporphyrin systems **29A,B** (Figure 29).

The influence of the resorcin[4]arene fragment on the porphyrin fluorescence was investigated by adding four structurally different fluorescence quenching quinones—benzoquinone (BQ), 1,4-naphthoquinone (NQ), phenanthrenequinone (PAQ) and dichlorodicyanobenzoquinone (DDQ)—to these porphyrin-resorcin[4]areneconjugates.

**Figure 30:**
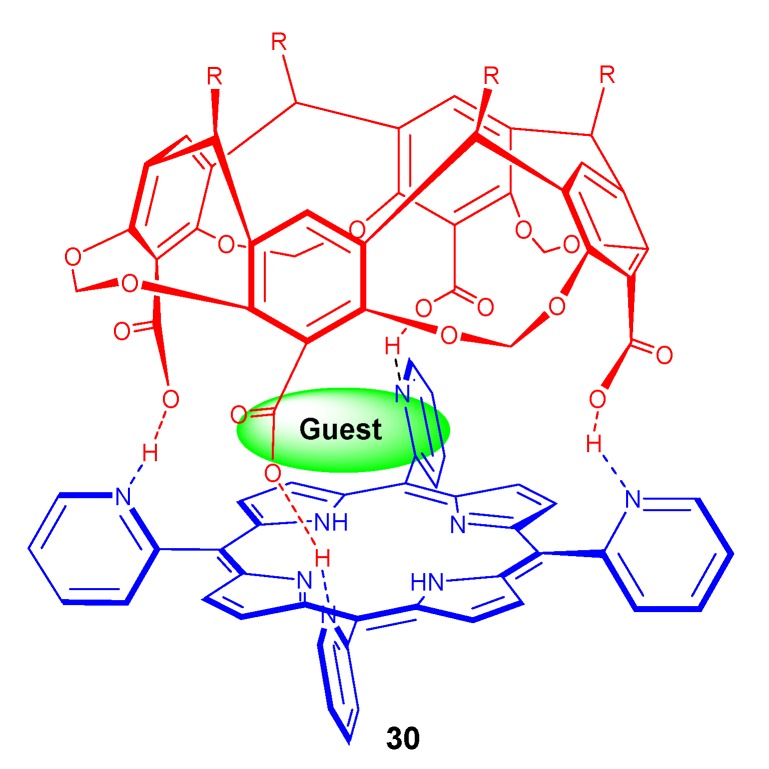
Small molecule encapsulation within the hydrogen bonded capsule (**30**).

It was observed that at low concentration of quinones, the fluorescence quenching of the porphyrin considerably enhanced by the attachment of resorcin[4]arene. The flexible unfirm resorcin[4]arene system **29B** (Figure 29) exhibited more fluorescence quenching compared to the rigid counterpart **29A** (Figure 29) when bulkier quinones are present which indicate the easy encapsulation of the bigger quinones within this flexible molecular system. At the same time when small sized benzoquinone was present, both rigid and flexible resorcin[4]arene-porphyrin system showed almost same fluorescence quenching. Nakazawa *et al*. synthesized a stable, quadruple hydrogen bonded capsule **30** (Figure 30) from tetracarboxy resorcinarene and 2-pyridylporphyrins [131]. This capsule is reported to encapsulate small molecules of various sizes from methane to cyclopentane. Various kinetic and thermodynamic parameters related to the process of encapsulation of small hydrocarbons into a resorcinarene-porphyrin conjugate and its guest exchanges rates has also been reported by the same group [132].

**Figure 31:**
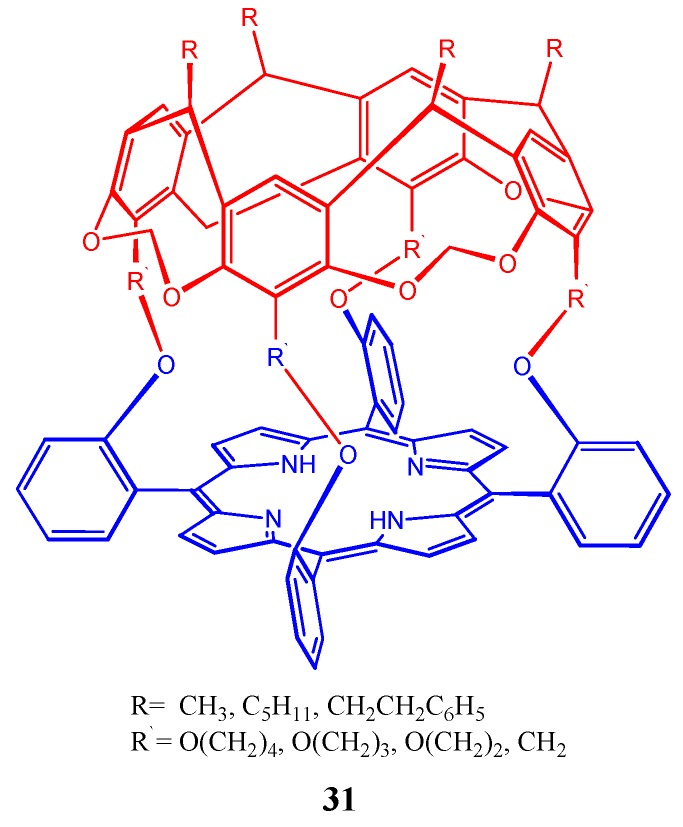
Representation of the resorcin[4]arene-capped porphyrin capsule (**31**).

Synthesis of novel resorcin[4]arene-capped porphyrin capsules **31** (Figure 31)was reported by McKay and coworkers [133].

They used microwave (MW) irradiation (in conjunction with Adler conditions) to make this important capsule and observed significant improvement in product yield relative to the traditional refluxing protocol.

Self-assembled (1:1) supramolecular resorcinarene-Zn porphyrin capsules containing coordinated bifunctional ligands such as 4,4'-bipyridine were synthesized by Stefanelli *et al*. [134]. The formation of these capsules depends on key structural factors, such as the size of the cavity, and the possibility of the onset of hydrogen bonds, π-π and π-cation interactions. The same preparative protocol is extended with chiral bifunctional ligands, such as (+)-cinchonine and (−)-cinchonidine, and cinchona alkaloid derivatives and resulted in the achievement of supramolecular structures with chiral cavities, whose configuration is dependent on the asymmetry of the bound ditopic ligand.

## 5. Conclusions

Cyclodextrin/calixarene/resorcinarene macrocyles can be used as preorganized three dimensional molecular receptors and functional materials by combination with porphyrins or metalloporphyrins. Owing to the cooperative contribution of the structural units, such molecular assemblies find unique application in catalysis, sensor devices, light harvesting or model structures for biomimetic research. The forthcoming research on these porphyrin incorporated molecular receptors is expected show an exponential growth because we are at a stage when researchers around the World are actively involved in biomimetic studies and nanosynthesis.
